# Relating genes to function: identifying enriched transcription factors using the ENCODE ChIP-Seq significance tool

**DOI:** 10.1093/bioinformatics/btt316

**Published:** 2013-06-03

**Authors:** Raymond K. Auerbach, Bin Chen, Atul J. Butte

**Affiliations:** Division of Systems Medicine, Department of Pediatrics, Stanford University School of Medicine, 1265 Welch Road, Room X-163 MS-5415, Stanford, CA 94305, USA

## Abstract

**Motivation:** Biological analysis has shifted from identifying genes and transcripts to mapping these genes and transcripts to biological functions. The ENCODE Project has generated hundreds of ChIP-Seq experiments spanning multiple transcription factors and cell lines for public use, but tools for a biomedical scientist to analyze these data are either non-existent or tailored to narrow biological questions. We present the ENCODE ChIP-Seq Significance Tool, a flexible web application leveraging public ENCODE data to identify enriched transcription factors in a gene or transcript list for comparative analyses.

**Implementation:** The ENCODE ChIP-Seq Significance Tool is written in JavaScript on the client side and has been tested on Google Chrome, Apple Safari and Mozilla Firefox browsers. Server-side scripts are written in PHP and leverage R and a MySQL database. The tool is available at http://encodeqt.stanford.edu.

**Contact:** abutte@stanford.edu

**Supplementary information:** Supplementary material is available at *Bioinformatics* online.

## 1 INTRODUCTION

Identifying gene or transcript signatures associated with development, disease and drug efficacy has been a focal point of biology and medicine since the release of the human genome sequence; however, focus has only recently shifted to relating these signatures to function on a genome-wide scale. Thanks to next-generation sequencing assays such as ChIP-Seq that query an entire genome, transcription factor-binding sites that may explain the underlying biochemical function behind these signatures can be identified (Johnson *et al.*, 2007; Robertson *et al.*, 2007). The ENCODE Consortium, in particular, has spent over US$123 million producing and analyzing functional assays for public use. As part of this effort, ENCODE has generated 843 ChIP-Seq experiments (including histone modifications and control experiments) across >90 cell lines from various tissues, treatments and conditions (ENCODE Project Consortium *et al.*, 2012). Although ENCODE ChIP-Seq experiments represent an invariable treasure trove of data to intersect against gene/transcript signatures and identify enriched transcription factors (TFs), the Consortium does not provide a simple tool for this purpose short of downloading and parsing each results file individually. Other tools, such as Cscan, have tried to fill this void, but their configuration options focus only on transcription start sites (Zambelli *et al.*, 2012). Additionally, Cscan uses the set of all genes from the entire human genome as a background set, rendering it impractical for signatures derived from expression microarrays or from array-capture–based sequencing experiments. We present the ENCODE ChIP-Seq Significance Tool, a simple, flexible single-page web application that addresses these gaps in existing tools, leverages public ChIP-Seq data from the ENCODE Production Phase and provides biomedical researchers the ability to conduct comparative analyses with their list of gene/transcript signatures.

## 2 DESCRIPTION

### 2.1 Underlying database

The ENCODE ChIP-Seq Significance Tool leverages a MySQL database of official, unified peak calls from 708 ENCODE ChIP-Seq non-histone and non-control experiments, encompassing 220 transcription factor and treatment combinations across 91 cell types. We first represent each called peak by the genomic position of its apex to minimize the effect of broader peak shapes biasing our database. The significant peak calls and the apex positions are those officially released by ENCODE for unrestricted public use. Using all protein-coding genes and transcripts as well as pseudogenes identified in the Gencode v15 annotation along with corresponding IDs and symbols from Entrez, Ensembl, HAVANA and the HUGO Gene Nomenclature Committee, we intersected the positions of all ChIP-Seq peak apexes from each experiment against the start and end positions of each gene, identifying the closest peak to the transcription start site (TSS) and transcription termination site (TTS). These values are recorded in the database for each gene/factor/cell line combination. Peak call files targeting the same combination of factor and cell line were pooled provided that the antibody target, cell treatments and binding context were the same. For example, experiments using different antibodies targeting the same carboxy-terminal domain repeat in the large subunit of RNA polymerase II to identify transcription initiation (e.g. Covance MMS-126 R and abcam ab5408) were pooled, but these experiments were kept separate from experiments using the abcam ab5095 antibody targeting the phosphorylated serine-2 of the same repeat to identify a stalled polymerase. Experiments using cells subjected to different treatments were also kept separate (e.g. untreated cells versus cells stimulated with interferon-γ).

### 2.2 ENCODE ChIP-Seq significance tool

**Fig. 1.**
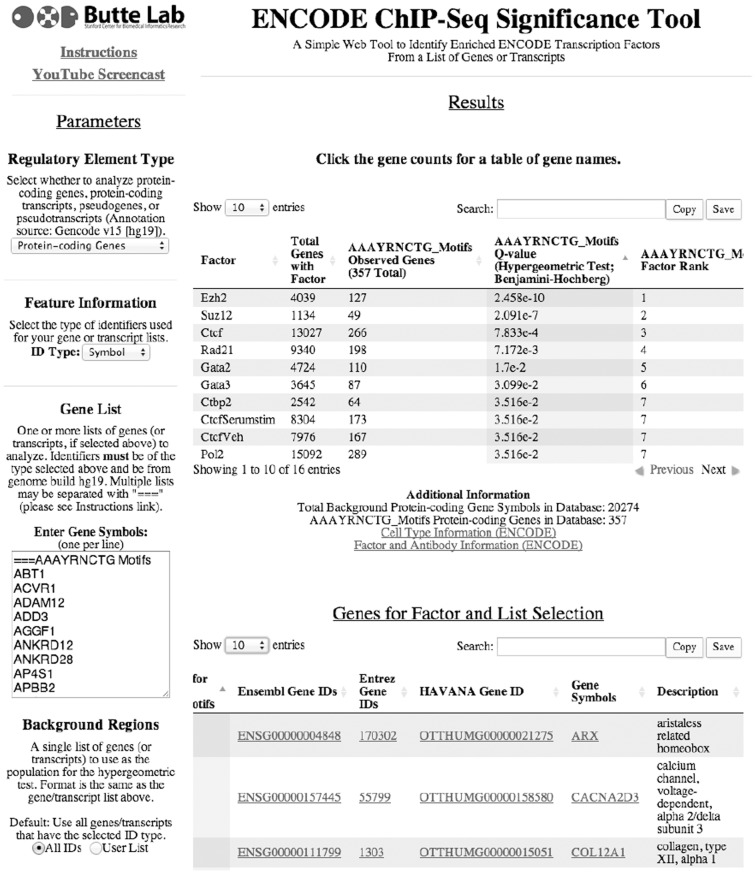
The ENCODE ChIP-Seq significance tool after a query

The ENCODE ChIP-Seq Significance Tool is a single-page web application that identifies enriched TFs in gene or transcript lists and presents the separate results from each list in a unified view (Fig. 1). The user begins by defining parameters including the gene/transcript ID system (Ensembl, Entrez, HAVANA or gene symbol from hg19), the feature type to use as the center of the window (TSS/5′-end, TTS/3′-end or the entire gene/transcript body), as well as the upstream and downstream analysis window size in increments of 500 bp. A gene list can be compared with the union of any combination of ChIP-Seq experiments in ENCODE Tier 1, 2 and 3 cell lines that comprise the ENCODE unrestricted dataset. For each unique TF/treatment combination, the ENCODE ChIP-Seq Significance Tool queries our database to identify the number of genes in each gene list with at least one TF peak apex in the selected window around the TSS, TTS or gene body. Enrichment scores are calculated using a one-tailed hypergeometric test followed by multiple hypothesis correction using the FDR method (Benjamini and Hochberg, 1995; Supplementary Material). Enrichment scores and gene counts for each TF across each list are shown in a table that can be saved, printed or copied to the clipboard directly from the tool. We also allow the user to specify a custom background list, allowing our tool to be applied to microarray data and other data not generated from genome- or transcriptome-wide assays.

In addition to identifying enriched TFs, researchers will also want to identify the specific genes that have a binding site for a particular transcription factor. By clicking on any cell in the results table, the user can retrieve a second table listing the gene IDs, associated metadata and links to external resources for each column and row combination. These tables can be copied to the user’s clipboard or saved to a file from within the web application.

### 2.3 Advantages over existing tools

Our curated database of ENCODE ChIP-Seq peaks coupled with our web interface offers several distinct advantages. First, existing tools tend to limit analyses to transcription start sites, but ENCODE data also include chromatin remodelers, DNA repair proteins and other classes of DNA-binding proteins that exhibit more promiscuous binding patterns. By offering the ability to select whole body or TTS analyses in addition to TSS analyses, we give researchers the flexibility to explore a wider range of biological questions. Existing tools also tend to restrict researchers to a limited range of window sizes near TSSs (e.g. ±1 kb). Our tool allows researchers to select a window size up to 5000 bp (in 500 bp increments) on each side of the TSS, TTS or gene/transcript body. Protein-coding genes/transcripts are also separated from pseudogenes in our underlying annotation. Additionally, we allow researchers to supply a custom background list, extending the application of our tool to signatures derived from methods that do not query the entire genome. Finally, comparative analysis between gene lists is common in many biomedical data analyses, but existing web tools often present comparisons one list at a time. For a researcher with multiple gene lists, this often means undertaking a time-consuming process of processing, saving and collating multiple comparisons manually. The ENCODE ChIP-Seq Significance Tool allows researchers to enter multiple lists in a single query, calculates significance scores for each list, and presents the separate results in a unified table to reduce both analysis time and the potential for human error.

## 3 RESULTS AND DISCUSSION

The ENCODE ChIP-Seq Significance Tool can be applied to a wide range of biological questions. We discuss two examples in the Supplementary Material: identifying the functional context of an unknown sequence motif and uncovering possible mechanisms of action for the drug dexamethasone. In the first example, we find that the unknown sequence motif appears in many promoter regions that are enriched for Polycomb group proteins, indicating genes that are likely repressed or that require strict regulation of chromatin structure. The unknown motif may even represent a binding site for a different Polycomb group protein. The second example shows that glucocorticoid receptor (Gr) is the most enriched TF among genes that are significantly upregulated after treatment with dexamethasone, an agonist of Gr. Most of the observed Gr-binding genes are related to inflammatory and immune disease, demonstrating that dexamethasone may act as an anti-inflammatory and an immunosuppressant mainly by targeting Gr-regulated, disease-related genes. This analysis helps identify the possible Gr-binding genes responsible for the drug action (e.g. the gene NFKBIA).

Please see the Supplementary Material for additional information about tool usage and features.

## Supplementary Material

Supplementary Data
