# Supplementary material for: Relating genes to function: identifying enriched transcription factors using the ENCODE ChIP-Seq significance tool
Source: Bioinformatics. 2013 Jun 3;29(15):1922–4. doi: 10.1093/bioinformatics/btt316 (PMC3712221; doi:10.1093/bioinformatics/btt316)
Supplement: Supplementary Data [file supp_29_15_1922__index.html]

Relating genes to function: identifying enriched transcription factors using the ENCODE ChIP-Seq significance tool — Relating genes to function: identifying enriched transcription factors using the ENCODE ChIP-Seq significance tool — Supplementary Data 

# Relating genes to function: identifying enriched transcription factors using the ENCODE ChIP-Seq significance tool

## Supplementary Data

files

**Files in this Data Supplement:**

- Supplementary Data - doc file
