# Supplementary material for: Relating genes to function: identifying enriched transcription factors using the ENCODE ChIP-Seq significance tool
Source: Bioinformatics. 2013 Jun 3;29(15):1922–4. doi: 10.1093/bioinformatics/btt316 (PMC3712221; doi:10.1093/bioinformatics/btt316)
Supplement: Supplementary Data [file supp_btt316_ENCODEQT_Supplement-RevisedNoTrackChanges.doc]

**ENCODE ChIP-Seq Significance Tool**

**Instructions, Use Cases, and Other Supplemental Information**

Raymond Auerbach (rauerbac@stanford.edu)

Butte Laboratory, Stanford University

Table Of Contents

Introduction [1](#__RefHeading___Toc229719592)

Advantages of Our Tool [2](#__RefHeading___Toc229719593)

The Interface [3](#__RefHeading___Toc229719594)

Description of Parameters [4](#__RefHeading___Toc229719595)

Regulatory Element Type and Feature Information [4](#__RefHeading___Toc229719596)

Gene or Transcript Lists [4](#__RefHeading___Toc229719597)

Entering multiple lists and naming lists [4](#__RefHeading___Toc229719598)

Background Regions [5](#__RefHeading___Toc229719599)

Analysis Window Parameters [5](#__RefHeading___Toc229719600)

Cell Lines [5](#__RefHeading___Toc229719601)

The Results Table [6](#__RefHeading___Toc229719602)

The Gene (or Transcript) Symbol Table [7](#__RefHeading___Toc229719603)

How is the Significance Calculated? [7](#__RefHeading___Toc229719604)

Use Cases [8](#__RefHeading___Toc229719605)

Functional Genomics Use Case [8](#__RefHeading___Toc229719606)

Drug Mechanism of Action Use Case [10](#__RefHeading___Toc229719607)

Frequently Asked Questions [12](#__RefHeading___Toc229719608)

Platforms Supported [14](#__RefHeading___Toc229719609)

Contact Information [14](#__RefHeading___Toc229719610)

Other Information and Acknowledgements [14](#__RefHeading___Toc229719611)

References [14](#__RefHeading___Toc229719612)

# Introduction

Biological analysis has shifted from identifying genes to mapping these genes to biological function. The ENCODE Project has generated hundreds of ChIP-Seq experiments for public use, spanning over 220 transcription factor and cell treatment combinations over 91 different cell lines (ENCODE Project Consortium, 2012; Land*t et a*l.). However, there are few simple tools available for biomedical scientists to analyze these data effectively and even these tools are tailored to explore a limited range of biological questions or require re-analysis of raw data. We present the ENCODE ChIP-Seq Significance Tool, a flexible web application that enables simple, comparative analyses by leveraging public ENCODE data to identify enriched transcription factors. Our tool is located at [http://encodeqt.stanford.edu](http://encodeqt.stanford.edu/).

# Advantages of Our Tool

Although there are other tools that can be used to analyze ChIP-Seq data given a gene list [CScan (Zambelli *et al.*, 2012) and oPOSSUM (Ho Sui *et al.,* 2007)] or a position-weight matrix for a sequence motif [LASAGNA-Search (Lee and Huang, 2013)], the ChIP-Seq TF Significance Tool offers users additional flexibility currently lacking in other ENCODE ChIP-Seq analysis applications. These features include:

- The ability for researchers to enter multiple gene or transcript lists at a time, greatly reducing the amount of analysis time and the potential for human error inherent when rerunning a tool multiple times. Results for multiple lists are also displayed in the same table, allowing for easy comparison.
- The ability for researchers to provide their own custom list of background genes or transcripts. In this manner, our tool can be used to query gene or transcript lists derived from expression microarrays, next-generation sequencing strategies that incorporate array-capture methods, or other assays that do not query the entire genome/transcriptome.
- Researchers can select any value for the analysis window from 500-5000 base pairs (bp) from the selected feature in either the upstream or downstream direction (0-5000 bp for gene/transcript body analysis) in 500 bp increments.
- Researchers are not limited to analyzing transcription factors from the perspective of the transcription start site.

In short, the ENCODE ChIP-Seq Significance Tool allows researchers to customize their analysis to better suit the questions they wish to explore. To illustrate some examples of where these features will prove useful, consider a transcription factor of interest that may be known *a priori* to bind upstream of a transcription start site (TSS). Using our tool, the analysis window can be set to include a greater area upstream of the TSS vs. downstream of the TSS.

Providing researchers the opportunity to use the entire gene/transcript body, the transcription start site, or the transcription termination site as the center of the analysis window allows for a broader range of biological questions to be explored, unlocking the full potential of ENCODE ChIP-Seq data. For example, consider the case of experiments using the “Pol2s2” antibody that are available as part of the public release (antibody information at <http://www.abcam.com/RNA-polymerase-II-CTD-repeat-YSPTSPS-phospho-S2-antibody-ChIP-Grade-ab5095.html>). Pol2s2 (or Pol2 phospho S2) experiments use an antibody that targets phosphorylated serine-2 in the carboxy-terminal domain repeat YSPTSPS of RNA polymerase II (RNAPII). When serine-2 becomes phosphorylated, RNAPII will stall. This can occur at intron/exon junctions and/or in genes that are poised for transcription until an external stimulus is received (e.g. heat shock). Because ChIP-Seq experiments using this antibody can produce peaks that occur anywhere across the gene body, using an analysis tool that limits the search space to only the transcription start site will cause researchers to miss important trends. By using the gene body feature of our tool, researchers could identify an enrichment of genes that show RNAPII stalling that would be missed by a TSS-centric analysis. These benefits stretch beyond just RNAPII, as ENCODE also includes antibody targets such as components of chromatin remodelers (Ini1, Brg1, BAF155, BAF170) and DNA damage repair proteins (Rad21) that also benefit from analyzing features beyond the transcription start site.

Our goal in designing the ENCODE ChIP-Seq Significance Tool is to provide researchers with as much flexibility as possible to ask the questions that they want to ask, rather than limit researchers by offering a narrow range of parameters to customize (e.g. ±1kb from a TSS) or require the use of the entire genome/transcriptome as a background set for statistical analysis.

# The Interface


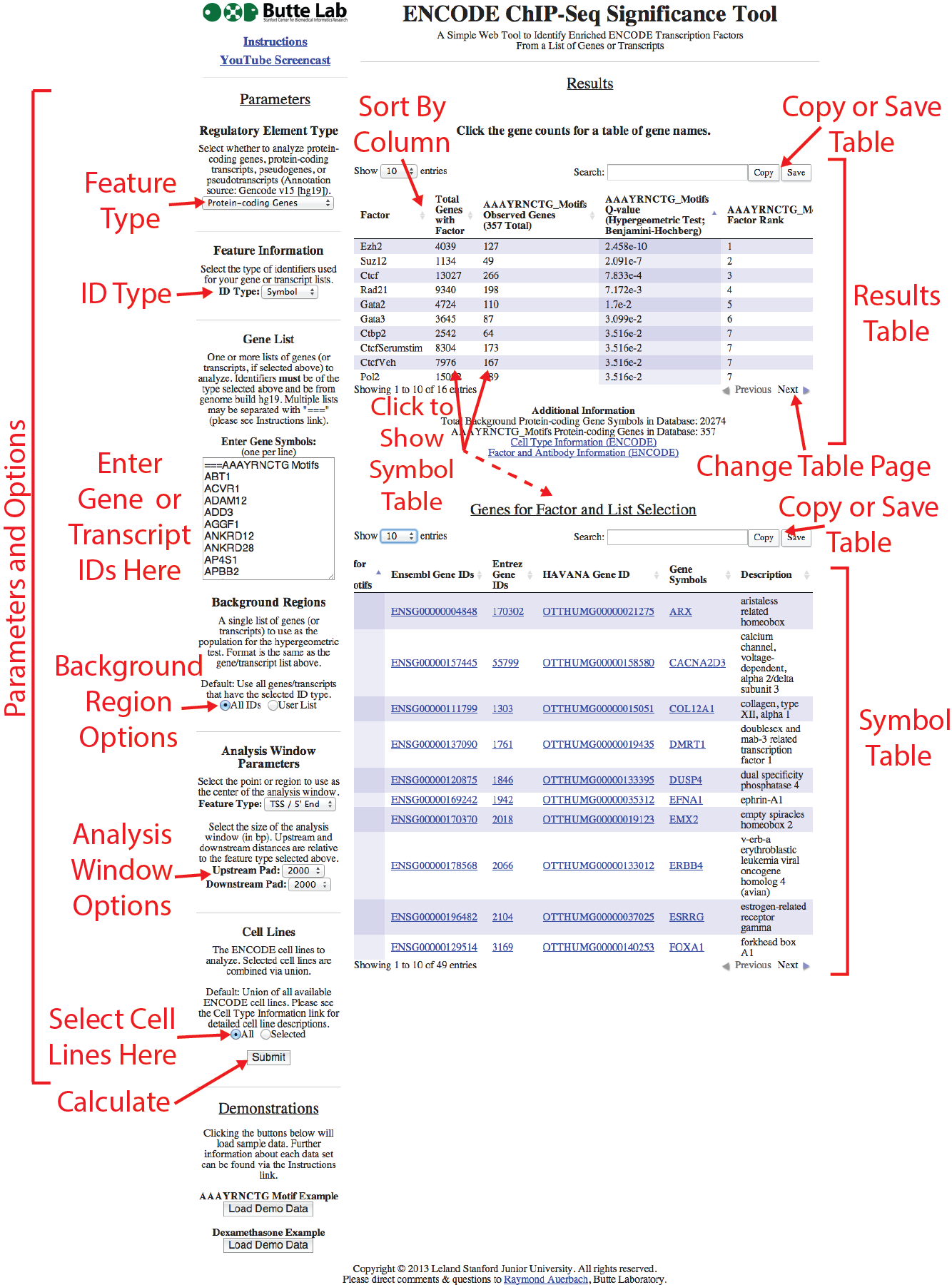


Figure S1: The interface of the ENCODE ChIP-Seq Significance Tool.

# Description of Parameters

Several parameters can be set to customize queries, including:

## Regulatory Element Type and Feature Information

**Regulatory Element Type** – The type of regulatory elements to target. Choices include protein-coding genes, protein-coding transcripts, pseudogenes, and transcripts from transcribed pseudogenes (or pseudotranscripts). Categories are based upon the Gencode v15 human genome annotation (Harro*w et a*l.).

**ID Type** – The ID system used by the gene/transcript list. The tool accepts gene and transcript IDs from Ensembl, Entrez and HAVANA as well as symbols from the HUGO Gene Nomenclature Committee (HGNC). All supported symbols are based upon human genome build hg19.

## Gene or Transcript Lists

Gene or transcript lists should be entered into text area designated, using one line per identifier. Accepted identifiers for genes include Ensembl Gene IDs (e.g. ENSG00000105991), Entrez Gene IDs (e.g. 3198), HAVANA Gene IDs (OTTHUMG00000023207) or HGNC gene symbols (e.g. HOXA1). Accepted identifiers for transcripts include Ensembl Transcript IDs (ENST00000343060), HAVANA transcript ID (OTTHUMT00000358454), or transcript symbols (HOXA1-001). IDs and symbols used should be from human genome version hg19. Different identifiers should **not** be mixed.

### Entering multiple lists and naming lists

The ENCODE ChIP-Seq Significance tool can analyze multiple gene or transcript lists in parallel as well as use custom names for each list (e.g. Overexpressed genes, Repressed genes, etc.). To name a list, preface it with the line “===<list title>”. For example, to compare a list of expressed and repressed genes the following could be entered into the ID List text area:

===Expressed Genes

<ExpressedGeneID1>

<ExpressedGeneID2>

…

<ExpressedGeneIDn>

===Repressed Genes

<RepressedGeneID1>

<RepressedGeneID2>

…

<RepressedGeneIDn>

List statistics are calculated separately and are never pooled across lists. However, the results table will show columns for each list with the name provided. The table widget can be scrolled horizontally for larger tables. Note: List names **must** be provided for all lists when analyzing multiple lists. For a single list, a list name may be provided using the above syntax otherwise a default name of “List1” will be used.

## Background Regions

**All IDs** – Uses the set of all identified genes or transcripts for the selected ID system as the population (background) for the hypergeometric test. This option is appropriate for most gene or transcript lists derived from whole genome or whole transcriptome sequencing.

**User List** – This option will cause an additional text area to be displayed, which expects a list of gene or transcript identifiers following the format described in “Gene and Transcript Lists” above. These identifiers MUST be a superset of the identifiers contained in the Gene and Transcript List text area (otherwise an error listing the missing identifiers will be shown) and will be used as the population/background for the hypergeometric test. This option is appropriate for gene or transcript lists derived from methods that do not probe the entire genome/transcriptome, such as some expression microarrays or array capture-based sequencing methods. Note that only a single background list may be entered for use with all gene/transcript lists.

## Analysis Window Parameters

**Upstream Pad** – the number of nucleotides upstream of the selected feature type to use for the analysis window. Used for TSS and TTS feature types.

**Downstream Pad** – the number of nucleotides downstream of the selected feature type to use for the analysis window. Used for “TSS/5’ end” and “TTS/3’ end” feature types.

**Upstream/Downstream Pad** – the number of nucleotides to use for the analysis window in both the upstream and downstream directions. Used only for the “Whole Body” feature type. Example: Selecting the value “500” will set the analysis window to start 500 nucleotides upstream of the TSS/5’ end and end 500 nucleotides downstream of the TTS/5’ end.

**Feature Type** – The gene or transcript feature to use as the center of the analysis window. This option can be set to use the transcription start site (TSS)/5’ end, the transcription termination site (TTS)/3’ end, or the entire gene/transcript body (“whole body”). Example analysis windows for each choice are shown in Figure S2.

## Cell Lines

**All** – Uses the union of all ChIP-Seq experiments (by factor/treatment) across all ENCODE cell lines for analysis.

**Selected** – Reveals a dropdown box where one or more ENCODE cell lines can be selected. Analysis is performed on the union of ChIP-Seq experiments (by factor/treatment) conducted in the selected cell lines.


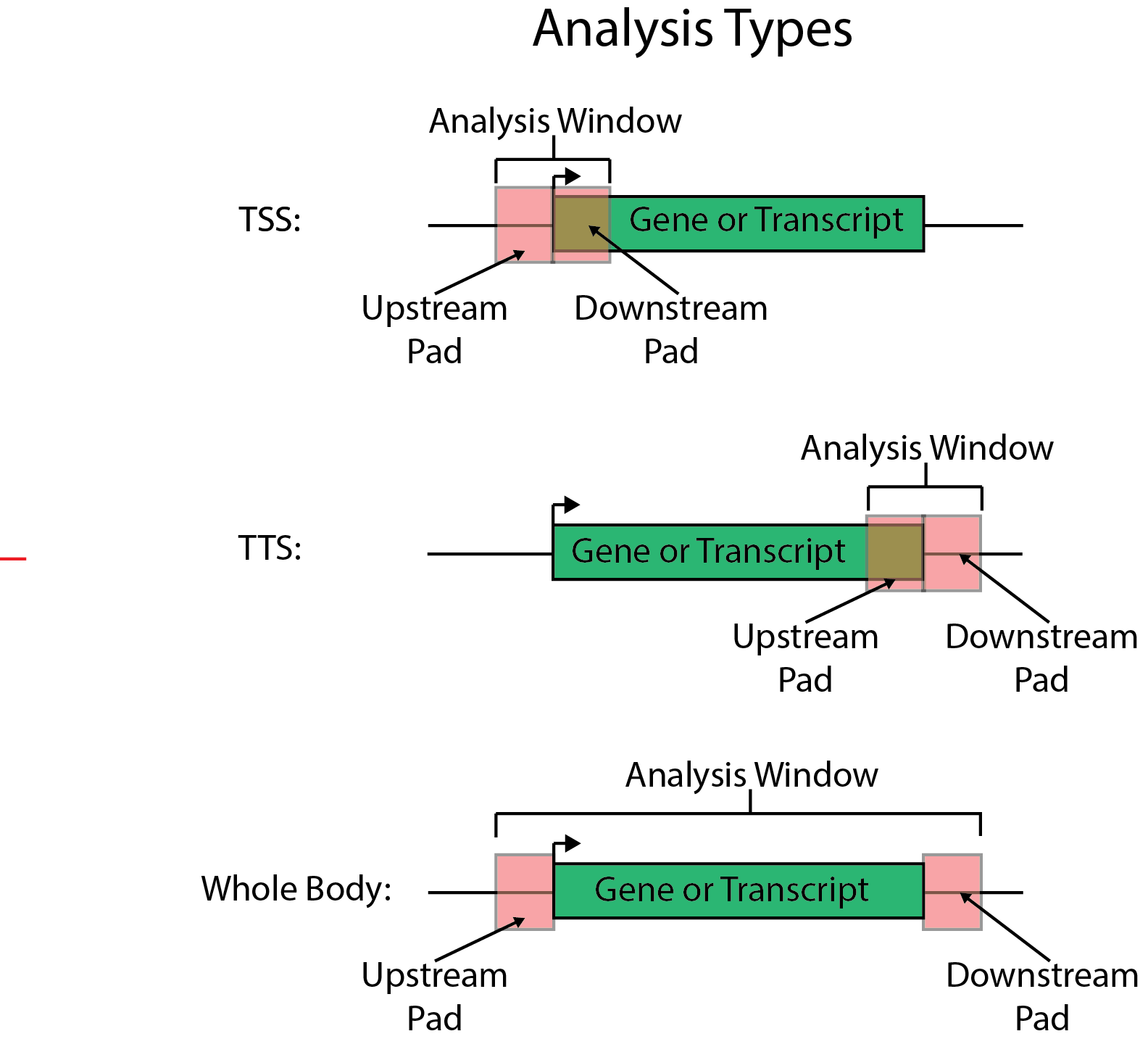


Figure S2: Gene feature analysis types.

# The Results Table

After all parameters are selected, clicking the “Submit” button will run the query. Results are returned in a table that can be sorted by clicking the appropriate column header, dynamically searched by factor name using the Search box, copied to the clipboard using the “Copy” button, printed using the “Print” button, or saved in PDF or CSV formats by using the “Save” button. The results table will always initially be sorted by the q-value of the first gene/transcript list in ascending order. A rank column for each list is also included in the rightmost columns of the table, ensuring that researchers always have access to the original sort order as they filter or search the results. Summary statistics including the number of identifiers matching genes/transcripts in our database appear below the results table. Additionally, links are provided to cell line and antibody descriptions from ENCODE’s main web portal.

Please note that the results table may span multiple pages and that the page selector is at the bottom-right of the table. The number of records displayed at once can also be changed used the selection box in the upper left of the table. If multiple gene lists were provided, scrolling the table widget to the right will reveal the additional columns.

# The Gene (or Transcript) Symbol Table

By clicking any value in the “Observed Genes” (or “Observed Transcripts”) columns in the Results table, an additional table will be created showing the genes or transcripts from the corresponding list that have a ChIP-Seq peak apex within the selected parameters. This list can be copied to the clipboard or saved to a local file by selecting the appropriate button at the top of the table. For all queries, the original identifier is displayed as well as the corresponding Ensembl, Entrez (for genes), and HAVANA IDs as well as the official HUGO Gene Nomenclature Committee (HGNC) symbol. Clicking an ID hyperlink will open the gene/transcript information page from the appropriate data source (Ensembl, NCBI, or HAVANA) in a new browser tab. Clicking on a gene or transcript symbol will open the corresponding symbol search page in the GeneCards database.

The same functionality and metadata also applies when selecting any total from the “Total Genes with Factor” column, except that **all** genes/transcripts with peaks matching the selected criteria in our database will be returned regardless of the provided gene lists.

As with the Results table, please note that the Gene Symbol table may span multiple pages and that the page selector is at the bottom-right of the table. The number of records displayed at once can also be changed used the selection box in the upper left of the table.

# How is the Significance Calculated?

Transcription binding significance is calculated using a hypergeometric test followed by multiple hypothesis correction using the method of Benjamini and Hochberg (also known as the false discovery rate, or FDR, method). The q-value calculated by our program is defined as the minimum false discovery rate at which a transcription factor can still be considered significant. Only transcription factors with a q-value < .05 will be reported.

For all statistical calculations, the ENCODE ChIP-Seq Significance Tool leverages the R Project. The hypergeometric test uses the p.hyper function (see the description at <http://stat.ethz.ch/R-manual/R-patched/library/stats/html/Hypergeometric.html>) with the following parameters:

- **q:** The number of genes/transcripts in the user-provided gene/transcript list with a peak apex for a given transcription factor within the analysis window.
- **m:** the total number of genes/transcripts in the population with a peak apex for a given transcription factor within the analysis window. The population is either all genes/transcripts in our database or the intersection of all genes/transcripts in our database with the user-provided background list, depending upon the parameters selected.
- **k:** the total number of genes/transcripts in the user-provided gene/transcript list that were also analyzed (i.e. the number of genes/transcripts in the list that are also in our database).
- **n:** the total number of genes/transcripts in the population without a peak apex for a given transcription factor within the analysis window. The population is either all genes/transcripts in our database or all genes/transcripts in our database from the user-provided background list, depending upon the parameters selected.
- **lower.tail:** FALSE. Calculates the probability that a transcription factor is enriched compared to the expected value.

R calculates the p-values for enrichment are calculated using a one-tailed hypergeometric test according to the following formula:


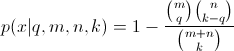


After the p-values are calculated for all transcription factor/treatment combinations for a given gene/transcript list, final q-values are calculated using a Benjamini-Hochberg multiple hypothesis correction (Benjamini and Hochberg, 1995). This correction is implemented by the R function “p.adjust” using the “BH” method parameter. Additional details about this function can be found at (<http://stat.ethz.ch/R-manual/R-devel/library/stats/html/p.adjust.html>). These final, Benjamini-Hochberg corrected q-values are shown in the results table.

# Use Cases

## Functional Genomics Use Case

**Identifying TF binding trends in promoters containing the AAAYRNCTG motif**

Note: By clicking the appropriate “Load Demo Data” button in the Demonstrations section of the web tool, the genes and parameters described in this use case will be set automatically.

Conserved DNA sequence motifs are present throughout the human genome, many of which may bind a transcription factor or other substrate. Identifying a binding partner requires biological validation in a traditional wet lab, but ENCODE ChIP-Seq data can be used to computationally generate hypotheses to test. To illustrate, we downloaded a public list of 378 genes from the Broad Institute that have an AAAYRNCTG motif within 2 kilobases (kb) of the transcription start site (<http://www.broadinstitute.org/gsea/msigdb/geneset_page.jsp?geneSetName=AAAYRNCTG_UNKNOWN>). The AAAYRNCTG motif is not associated with a known transcription factor. We set our parameters to use a window of ±2 kb from the transcription start site, mirroring the Broad Institute’s study. We also choose to look across all cell lines, as our gene list focuses on primary sequence data rather than tissue-based data. Running the ENCODE ChIP-Seq Significance Tool generated the results in Figure S3.

As shown in the results below, 355 of the 378 genes are in our database and were used for TF enrichment calculations. The promoter regions from this list are most enriched for the transcription factors Ezh2 and Suz12. Both Ezh2 and Suz12 are members of the Polycomb-group proteins that remodel chromatin to promote epigenetic silencing of genes. At best, it is possible that our unknown motif may bind another member of the Polycomb complex or a transcription factor that is also related to this process. At worst, it is possible that Polycomb-group proteins are silencing these genes and that the maintenance of the underlying chromatin structure is particularly important to this set of genes. These hypotheses can be further refined using other computational methods (GO analysis of the gene list, etc.) and tested in a traditional lab setting. For example, a researcher might next look at RNAseq data to examine the conditions under which these genes may be silenced. This is just one example of how ENCODE data and the ENCODE ChIP-Seq Significance Tool can be used to probe existing data sets.


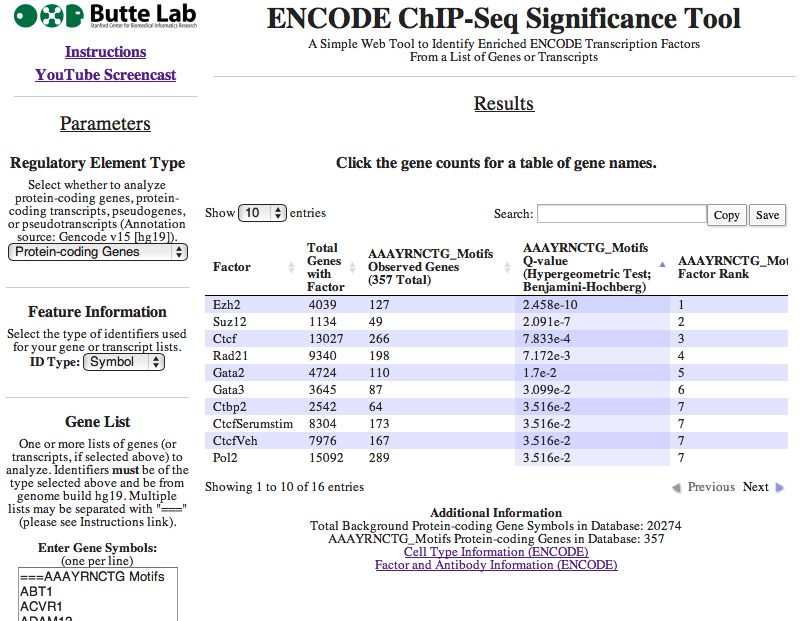


Figure S3: Results from functional genomics use case, sorted by q-value. Clicking on any cell total will show the corresponding gene symbols in a separate table.

## Drug Mechanism of Action Use Case

**Exploring potential mechanisms of action for Dexamethasone**

Note: By clicking the appropriate “Load Demo Data” button in the Demonstrations section of the web tool, the genes and parameters described in this use case will be set automatically.

We collected 22 microarray studies from GEO that provided gene expression profiles from human cells upon treatment with Dexamethasone (an anti-inflammatory and immunosuppressant) versus treatment with vehicle (or untreated), comprising 252 samples. A meta-analysis was then performed (Morga*n et a*l., 2010) and 46 genes were significantly expressed (FDR<0.05) (table S1). Using these genes to query the ENCODE ChIP-Seq Significance Tool (feature type: TSS /5’ End, upstream pad: 1000, downstream pad: 1000), we found that glutocorticoid receptor (Gr) appeared among the most enriched transcription factors among 217 entries (table S2). Upon further examination of the observed Gr binding genes, we could identify a few genes (e.g., BIRC3, NFKBIA) linking this drug to inflammatory and immune diseases. The same techniques can be applied to other drug studies to identify potential drug targets, to better understand how a drug may function, and to identify possible new targets and applications for existing drugs.

Table S1: Significantly expressed genes of Dexamethasone and those mapping to Gr binding genes.

| **GeneID** | **Symbol** | **q_value** | **up_down** | **Mapped to  Gr-binding genes?** |
| --- | --- | --- | --- | --- |
| 330 | BIRC3 | 2.47E-02 | up | yes |
| 687 | KLF9 | 1.93E-02 | up | no |
| 1052 | CEBPD | 0.00E+00 | up | yes |
| 1831 | TSC22D3 | 2.58E-04 | up | no |
| 1843 | DUSP1 | 3.76E-04 | up | yes |
| 2180 | ACSL1 | 5.23E-03 | up | no |
| 2289 | FKBP5 | 6.40E-04 | up | yes |
| 2353 | FOS | 1.21E-02 | up | yes |
| 2752 | GLUL | 1.10E-07 | up | yes |
| 2766 | GMPR | 1.86E-03 | up | no |
| 4792 | NFKBIA | 1.40E-04 | up | yes |
| 4837 | NNMT | 7.43E-03 | up | yes |
| 5187 | PER1 | 5.18E-08 | up | yes |
| 5406 | PNLIP | 5.83E-07 | up | no |
| 5997 | RGS2 | 1.33E-02 | up | no |
| 6446 | SGK1 | 5.03E-03 | up | no |
| 7278 | TUBA3C | 8.79E-04 | up | no |
| 7538 | ZFP36 | 6.20E-03 | up | yes |
| 7704 | ZBTB16 | 3.75E-05 | up | no |
| 8660 | IRS2 | 7.12E-03 | up | no |
| 27244 | SESN1 | 7.68E-05 | up | no |
| 28231 | SLCO4A1 | 7.25E-03 | up | yes |
| 28984 | C13orf15 | 3.61E-02 | up | no |
| 51655 | RASD1 | 1.65E-04 | up | yes |
| 54541 | DDIT4 | 7.70E-03 | up | yes |
| 55224 | ETNK2 | 1.35E-05 | up | yes |
| 55273 | TMEM100 | 6.02E-04 | up | no |
| 57488 | ESYT2 | 2.17E-03 | up | yes |
| 79627 | OGFRL1 | 3.59E-02 | up | yes |
| 83716 | CRISPLD2 | 1.91E-09 | up | no |
| 83733 | SLC25A18 | 3.71E-13 | up | no |
| 84913 | ATOH8 | 6.73E-04 | up | no |
| 115572 | FAM46B | 6.40E-04 | up | no |
| 116154 | PHACTR3 | 1.72E-05 | up | no |
| 117283 | IP6K3 | 5.14E-08 | up | yes |
| 122402 | TDRD9 | 4.49E-08 | up | no |
| 126695 | C1orf172 | 2.19E-04 | up | no |
| 153218 | SPINK13 | 5.54E-11 | up | yes |
| 157310 | PEBP4 | 4.92E-02 | up | yes |
| 199964 | TMEM61 | 1.75E-07 | up | no |
| 286887 | KRT6C | 4.50E-03 | up | no |
| 727751 | LOC727751 | 0.00E+00 | up | no |
| 6347 | CCL2 | 3.99E-10 | down | yes |
| 8870 | IER3 | 3.67E-03 | down | no |
| 10769 | PLK2 | 2.41E-02 | down | yes |
| 91523 | FAM113B | 3.53E-02 | down | no |

**Table S2: Significantly enriched TFs for the Drug Mechanism of Action Use Case (q < .05).**

| **Factor** | **Total Genes with Factor** | **List1 Observed Genes (44 Total)** | **List1 Q-value (Hypergeometric Test; Benjamini-Hochberg)** |
| --- | --- | --- | --- |
| Gr | 4366 | 20 | 3.437E-02 |
| Ctcf | 10865 | 35 | 3.437E-02 |
| Nelfe | 291 | 4 | 3.437E-02 |
| Rad21 | 7210 | 27 | 3.437E-02 |

# Frequently Asked Questions

**Q:** How does the ENCODE ChIP-Seq significance tool treat analysis windows that contain multiple peaks?

**A:** Analysis windows with multiple peaks are treated the same way as analysis windows with a single peak (that is, a transcription factor is deemed present for the hypergeometric test if at least one ChIP-Seq peak is present). The goal of our tool is to identify genes/transcripts that are enriched for binding of specific transcription factors. Multiple peaks can be called for a variety of different reasons ranging from the efficacy of an antibody in a particular cell line, the chromatin conformation at a particular locus and if it allows the antibody to access the transcription factor, the peak calling parameters used, and finally if there are actually multiple instances of binding by the same subunit. We anticipate our tool being used for hypothesis generation and that hypotheses will be validated using experimental methods. We feel that reporting any significant binding observed (as measured by a called peak in the ENCODE ChIP-Seq data) is consistent with this vision.

**Q:** Why did you choose to take the union of ChIP-Seq experiments across cell lines instead of the intersection?

**A:** Deciding whether to combine or split data is a central design choice made in many bioinformatics analyses and both approaches have their merits. In this case we chose to start with the union because we envisioned analytical questions like “show me the enriched TFs in all of the lung cancer cell lines available in ENCODE.” Also, for many ENCODE Tier II and Tier III data sets, the transcription factors available can be pretty disjoint (for example, a ChIP experiment targeting one member of a complex might be available in one cell line but not available in another). By taking the intersection, we worried that the absence of a significant TF in one cell line over another would implicitly suggest that a transcription factor was not significant in a particular cell line when in reality the data simply hasn’t been generated/released yet. As ENCODE fills in the gaps in these cell lines in their next phase, and if there is demand, we would consider adding an option for the intersection in our next version. Also as a side consideration, the output is kept much more clean for both the user and the developer by taking the union.

**Q:** Where is the data for modENCODE and ENCODE mouse?

**A:** The ENCODE ChIP-Seq Significance Tool uses peaks generated by the ENCODE Consortium using a uniform set of methods and control data sets. The modENCODE and ENCODE Mouse consortia have not released similar unified ChIP-Seq peak lists yet, although we expect that they will do so in the future. If there is user demand, we can include these additional organisms in our tool at that time.

**Q:** Which ENCODE ChIP-Seq peak calls were used to create this database?

**A:** The ENCODE Project has produced several different sets of peak call files during their existence. Our database is built upon the ENCODE Unified Peak Calls generated by the PeakSeq program and used in their September 2012 paper. To generate these files, the ENCODE Consortium re-scored all submitted ChIP-Seq experiments (which at that time had been scored by the different labs using a range of programs and parameters) and scored each against a common control data set using the PeakSeq and SPP peak calling programs (Rozowsk*y et a*l., 2009; Kharchenk*o et a*l., 2008). ENCODE further refined these peak lists by applying the Irreproducibility Discovery Rate Framework to keep only those peaks that were found to be reproducible between replicates and to automatically determine an appropriate peak calling threshold. Their workflow is detailed in the cited Nature paper as well as at [www.encodestatistics.org](http://www.encodestatistics.org/).

**Q:** The ENCODE paper cites more transcription factor experiments than we claim to have in our underlying database.

**A:** Our underlying database does not include ChIP-Seq experiments targeting histone modifications or experiments used as controls (e.g. those comprised of input DNA or using a non-specific antibody for ChIP). Our database only includes peak calls from ENCODE ChIP-Seq experiments targeting transcription factors.

**Q:** Does conducting an analysis on a gene list use all known transcripts for a gene or the annotated start and end coordinates for a gene?

**A:** Currently, choosing to conduct analyses on genes will use the coordinates *for the gene entry* according to the Gencode v15 annotation. Individual transcripts are not considered. To conduct analyses on a transcript level, a list of transcript IDs should be provided as input and the Regulatory Element Type setting should be set to protein-coding transcripts or pseudotranscripts.

# Platforms Supported

The ENCODE ChIP-Seq Significance Tool has been tested on the latest versions of Apple Safari, Google Chrome, and Mozilla Firefox.

# Contact Information

Questions and comments regarding the ENCODE ChIP-Seq Significance Tool can be directed to Raymond Auerbach at [rauerbac@stanford.edu](mailto:rauerbac@stanford.edu). Questions regarding individual ENCODE experiments can usually be answered by visiting the ENCODE portal (<http://encodeproject.org/ENCODE/>) or by contacting the appropriate consortium members. The ENCODE ChIP-Seq Significance Tool is not directly affiliated with the ENCODE Project.

# Other Information and Acknowledgements

The database that underlies the ENCODE ChIP-Seq Significance Tool was generated using only the PeakSeq ChIP-Seq unified peak calls included as part of the ENCODE Consortium paper released in September, 2012 (ENCODE Project Consortium, 2012). All underlying data sets are free to use without embargo or restriction and are provided in compliance with the ENCODE Data Use Policy.

The authors would like to thank the NHGRI and members of the ENCODE Consortium for providing a vast public data set that can be used to explore a wide range of scientific questions and for making these data publicly available.

# References

Benjamini,Y. and Hochberg,Y. (1995) Controlling the false discovery rate: a practical and powerful approach to multiple testing. *Journal of the Royal Statistical Society Series B,* **57**(1), 289-300.

The ENCODE Project Consortium. (2012) An integrated encyclopedia of DNA elements in the human genome. *Nature*, **489**, 57–74.

Harrow,J. *et al.* GENCODE: The reference human genome annotation for The ENCODE Project.

Ho Sui,S.J. *et al.* (2007) oPOSSUM: integrated tools for analysis of regulatory motif over-representation. *Nucleic Acids Res.* **35**, W245-52.

Kharchenko,P.V. *et al.* (2008) Design and analysis of ChIP-seq experiments for DNA-binding proteins. *Nat Biotechnol*, **26**, 1351–1359.

Landt,S.G. *et al.* (2012) ChIP-seq guidelines and practices of the ENCODE and modENCODE consortia. *Genome Res*, **22**(9), 1813-1831.

Lee,C. and Huang,C.H*.* (2013) LASAGNA-Search: an integrated web tool for transcription factor binding site search and visualization. *Biotechniques* **54**(3), 141-153.

Morgan,A.A. *et al.* (2010) Comparison of multiplex meta analysis techniques for understanding the acute rejection of solid organ transplants. *BMC Bioinformatics*, **11 Suppl 9**, S6.

Rozowsky,J.S. *et al.* (2009) PeakSeq enables systematic scoring of ChIP-seq experiments relative to controls. *Nat Biotechnol*, **27**, 66–75.

Zambelli,F. *et al.* (2012) Cscan: finding common regulators of a set of genes by using a collection of genome-wide ChIP-seq datasets. *Nucleic Acids Res.*, **40**, W510–515
